# The Efficacy of Immune Checkpoint Inhibitors in the EGFR Mutant and Wild‐Type Non‐Small Cell Lung Cancer Is Positively Associated With the Maturation and Abundance of Dendritic Cells

**DOI:** 10.1111/1759-7714.70049

**Published:** 2025-03-25

**Authors:** Fengqi Xiao, Yanguo Liu, Xiuwen Wang

**Affiliations:** ^1^ Department of Medical Oncology Qilu Hospital of Shandong University Jinan Shandong China

**Keywords:** dendritic cells, immune checkpoint inhibitors, non‐small cell lung cancer, TME

## Abstract

**Background:**

Dendritic cells (DCs) are known to be crucial in initiating immune responses, but their role in regulating immune checkpoint inhibitor (ICI) efficacy in EGFR mutant NSCLC remains unclear.

**Methods:**

Peripheral blood mononuclear cells (PBMCs) were co‐cultured with EGFR mutant cells to evaluate immune scores and DC maturation via high‐throughput sequencing. TIDE scores were used to predict the efficacy of ICI treatment. Gene set enrichment analysis (GSEA) was carried out on DCs to explore the signaling pathway changes underlying the diverse responses to ICIs.

**Results:**

A significant decrease in CD8+ T lymphocytes and cytotoxicity scores was found in EGFR mutant LUAD compared to wild‐type (*p* < 0.001). Three datasets (GSE135222, GSE126044, and GSE136961) showed that higher DC gene expression was associated with a more favorable response to ICIs (*p* = 0.028). The CSE241934 dataset showed that the number of conventional DC 1 (cDC1) was higher in the ICI‐sensitive group. The TIDE model suggested that cDC1 was associated with ICIs efficacy. However, GSE32863, GSE75037, and GSE72094 showed no differences in cDC subpopulations between EGFR mutant and wild‐type LUAD. EGFR mutant cells exhibited more suppression in the expression of HLA‐DR, CD40, CD83, and CD86 than the control group. The TIDE model suggested DC maturity was associated with ICI efficacy. GSE241934‐IIT showed that DC maturity was more abundant in the ICI‐sensitive group than that in the resistant group.

**Conclusions:**

Both the number and maturation capacity of DCs are positively correlated with ICI efficacy. The cause of poor ICI efficacy in EGFR mutant LUAD is more likely to be low DC maturity, not number, compared to EGFR wild‐type LUAD.

## Graphical abstract

Dendritic cells gene expression signature predicted ICIs efficacy. (A) IRF8 high‐expression group had low TIDE scores, indicating a predicted longer survival period following ICIs treatment. (B) CLEC9A high‐expression group had low TIDE scores, indicating a predicted longer survival period following ICIs treatment. (C–E) The stack diagram showed the cell composition of each sample, GSE135222 (*n* = 27), GSE126044 (*n* = 16), GSE136961 (*n* = 21). Each column in the above figure corresponds to each sample, and a different color indicates the constituent specific gravity of the cells in each sample. The dark blue is the component of DC cells in each sample. (F) The surv_cutpoint value. (G) Kaplan‐Meier survival analysis of the different groups of samples from GEO dataset, patients with high abundance of dendritic cell infiltration had a high therapeutic benefit heel from ICIs. (H)The number of cDC1 in GSE241934‐Real dataset between the sensitive group and resistant group follwing ICIs. (I) The number of cDC2 in GSE241934‐Real dataset between the sensitive group and resistant group follwing ICIs. (J) The number of pDC in GSE241934‐Real dataset between the sensitive group and resistant group follwing ICIs.

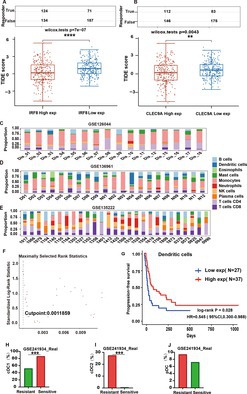

## 1 Introduction

According to the GLOBALCAN data published by the International Agency for Research on Cancer of the World Health Organization, lung cancer is the most common malignant neoplasm [1], classified into non‐small cell lung cancer (NSCLC) and small cell lung cancer (SCLC). NSCLC constitutes about 85% of cases, with lung adenocarcinoma (LUAD) as the most prevalent subtype, representing over 50% of cases. The prevalence of epidermal growth factor receptor (EGFR) mutations is 17% in Western populations but can reach 50%–60% in Asian populations [2, 3, 4, 5, 6]. Tyrosine kinase inhibitors (TKIs) are the primary therapeutic approach for advanced EGFR‐mutated LUAD [7, 8, 9]. Nonetheless, resistance to EGFR‐TKIs leads to disease progression in most patients. Conventional treatments, such as chemotherapy and anti‐angiogenesis therapy, have limited efficacy and provide only modest survival benefits in cases of resistance.

Immune checkpoint inhibitors (ICIs), specifically anti‐PD‐1/PD‐L1 agents, have shown significant therapeutic efficacy in advanced LUAD patients lacking EGFR mutations. However, the effectiveness of ICIs in EGFR mutant LUAD patients remains suboptimal. Initially, research focused on programmed death‐ligand 1 (PD‐L1) expression in EGFR mutant LUAD to understand resistance mechanisms to ICIs. Subsequent studies revealed higher PD‐L1 expression in EGFR mutant compared to EGFR wild‐type LUAD [10, 11]. Conversely, some researchers observed reduced PD‐L1 expression in EGFR mutant LUAD [12, 13]. These conflicting findings suggest that relying solely on PD‐L1 expression to explain ICI resistance in EGFR mutant LUAD is imprecise. Clinical studies have identified a subgroup of patients with low PD‐L1 expression who still respond to ICIs, indicating additional factors beyond PD‐L1 in ICI resistance mechanisms in EGFR mutant LUAD.

The interaction among cells in the tumor microenvironment (TME) significantly impacts the patient's response to therapy. Patients with EGFR mutation exhibit an immunosuppressed TME [14], characterized by low immunogenicity and lack of inflammation compared to wild‐type LUAD [12, 15]. A notable decrease in infiltrating CD8+ tumor‐infiltrating lymphocytes is observed in EGFR mutant LUAD, indicating potential factors hindering effector T‐cell induction and proliferation within the TME. The scarcity of CD8+ T cells, crucial for tumor cell elimination, may reflect TME immunosuppression, with the precise underlying mechanism remaining elusive. Initial studies have explored how EGFR mutant tumor cells create a suppressive TME. However, these studies have not examined the mechanisms in detail and require extensive research.

Dendritic cells (DCs) are essential components of the immune system in the TME, playing a critical role in initiating, regulating, and sustaining immune responses. Dysfunction of DCs in the tumor TME [16, 17], characterized by maturation disorders and phenotypic changes, hinders effective activation of CD8+ T cells. Restoring DC function to enhance T cell activation has emerged as a key research focus to improve the therapeutic outcomes of ICIs [18, 19, 20, 21]. Despite numerous studies supporting this approach, the underlying mechanisms remain to be fully elucidated and warrant further investigation. The key population of cells and mechanisms of how to reshape a weakly immunogenic, non‐inflammatory TME in EGFR mutant LUAD remain unidentified. It is crucial to pinpoint the key population of cells responsible for the immunosuppressive environment in EGFR‐mutated patients and elucidate how they promote inadequate immune responses and resistance to ICIs in this setting. This will be essential for developing more effective therapeutic strategies.

## 2 Materials and Methods

### 2.1 Cell Lines

The human non‐small cell lung carcinoma cell lines HCC827 and PC9 were purchased from the Cell Resource Center of the Chinese Academy of Sciences (Beijing, China) and cultured in RPMI‐1640 (Gibco) supplemented with 10% fetal bovine serum (FBS; Gibco) and 1% penicillin–streptomycin solution.

### 2.2 Peripheral Blood

Human whole peripheral blood mononuclear cells (PBMCs) were isolated using Lymphoprep (StemCell Technologies) in accordance with the manufacturer's instructions. This study was approved by the ethics committee for human studies, and all informed consent was obtained.

### 2.3 PBMC Isolation

Fresh anticoagulated whole blood was diluted with PBS. An appropriate amount of separation solution was added to a centrifuge tube, and then the diluted blood was spread on top of the separation solution. The tubes were then centrifuged for 20–30 min at 500–1000 g. After centrifugation, there was observable stratification between layers: the buffy coat layer between the plasma and the separation fluid was the lymphocyte layer. This layer was pipetted into a 15 mL centrifuge tube and washed with either 10 mL of PBS or cell washing solution before being centrifuged for 10 min at 250 g. The supernatant was then discarded before the remaining cells were resuspended in 5 mL of either PBS or cell washing solution and then centrifuged at 250 g for 10 min. The step of resuspending in 5 mL of either PBS or cell washing solution was repeated to discard the supernatant and resuspend the cells for later use.

### 2.4 mRNA‐Sequence

Total RNA was isolated using the RNeasy Mini Kit (Qiagen, Germany). The paired‐end libraries were synthesized using the TruSeq RNA Sample Prep Kit (Illumina, USA) following the TruSeq RNA Sample Preparation Guide. Briefly, mRNA molecules containing poly‐A were purified using magnetic beads attached to poly‐T oligonucleotides. After purification, mRNA was cleaved with divalent cations for 8 min at 94°C. The cleaved RNA fragments were then copied into first‐strand cDNA using reverse transcriptase and random primers. Second‐strand cDNA synthesis was conducted using DNA polymerase I and RNase H. The cDNA fragments underwent an end‐repair process, whereby a single “A'” base was added, and ligation of the adapters occurred. After the products were purified and enriched by PCR, the final cDNA library was created. The library construction and sequencing were carried out by Sinotech Genomics Co. Ltd. (Shanghai, China).

### 2.5 Analytical Methods Related to Bioinformatic Analysis

#### 2.5.1 Immune Score

RNA‐Seq data and clinical information for lung cancer were retrieved from The Cancer Genome Atlas (TCGA) dataset (https://portal.gdc.com) or the GEO database. Differences in the abundance of tumor‐infiltrating immune cells among sample groups were assessed using the immunedeconv R software package. The subsequent findings were visualized using the R (v 4.0.3) software packages ggplot2 and pheatmap.

#### 2.5.2 Prediction of ICI Therapy Response

RNAseq data (level 3) and relevant clinical data for LUAD were acquired from TCGA. The TIDE algorithm was utilized to forecast potential responses to immunotherapy, where elevated TIDE scores indicated diminished efficacy of ICI therapy and shorter post‐ICI survival. Prediction of potential ICI response was conducted using the TIDE algorithm [22].

#### 2.5.3 Single‐Cell Sequencing Analysis

Gene expression files were obtained from the GEO database (https://www.ncbi.nlm.nih.gov/geo/) and analyzed using the Seurat package to determine gene count, cell count, and mitochondrial content percentages. Low‐quality cells were removed during standard data preprocessing. Quality control metrics were visualized, data were filtered, batch effects were removed, highly variable genes were identified, principal component analysis was conducted, cell clusters were generated using the UMAP method, clusters were manually annotated, and differential gene expression across samples and cell populations was examined.

#### 2.5.4 LASSO Regression

The Least Absolute Shrinkage and Selection Operator (LASSO) regression technique was employed to develop a multi‐gene signature comprising Oncogenic Cell Genes (OCGs) for prognostic prediction in LUAD, utilizing the “glmnet” package within the R software suite. To enhance the reliability and objectivity of the analytical outcomes, a 10‐fold cross‐validation procedure was executed to ascertain the optimal lambda parameter, which corresponded to the minimum partial likelihood deviance.

#### 2.5.5 Gene Set Enrichment Analysis

The R packages limma (version 3.38.3) [23] and DESeq2 (version 1.22.2) [24] were employed to compute the differential expression t‐statistics for both microarray and RNA sequencing data. The resultant t‐statistics were utilized as input for the R function fgsea, which is implemented within the fgsea package (version 1.6.0), to conduct gene set enrichment analysis (GSEA). The signaling pathways from the Molecular Signatures Database (MSigDB) were utilized during the GSEA process [25].

### 2.6 Statistical Analysis

All data were expressed as mean ± SEM. GraphPad Prism 9.0 was used for statistical analysis. A minimum of three independent results from cell experiments were evaluated.  The differences between two groups were verified using unpaired Student's t‐test in high‐throughput sequencing analysis. The significance of two sample groups is tested by the Wilcoxon test, and that of three or more sample groups is tested by the Kruskal‐Wallis test in TIDE (Tumor Immune Dysfunction and Exclusion) algorithm and immune scores analysis. PFS between two groups was compared using the Kaplan–Meier survival analysis and log‐rank test. Results with two‐sided *p* < 0.05 were considered statistically significant.

## 3 Results

### 3.1 EGFR Mutant NSCLC Immune Checkpoint Inhibitors Were Poorly Effective Compared With Wild Type

Significant progress has been made in the treatment of advanced LUAD patients without EGFR mutation through the use of ICIs, particularly PD‐1/PD‐L1 monoclonal antibodies. Consequently, there has been a shift from single‐agent to combination therapy, now widely accepted as the standard treatment strategy. However, several studies have demonstrated the ineffectiveness of ICIs in patients with EGFR mutant LUAD (Table 1). The OAK study validated that Atezolizumab did not enhance overall survival (OS) in EGFR mutant patients (HR = 1.24, 95% CI 0.71–2.18). Notably, Atezolizumab outperformed docetaxel in patients with low or absent PD‐L1 expression, indicating the involvement of intricate mechanisms beyond PD‐L1 in the anti‐tumor efficacy of ICIs [26]. In the CheckMate 057 trial, encompassing 15% of patients with EGFR mutations, subgroup analyses revealed that the nivolumab monotherapy group did not exhibit superiority over docetaxel (HR = 1.18, 95% CI = 0.69–2.0). Within the Keynote 010 study, among patients with EGFR mutations who had received prior diverse treatments, no tendency toward greater OS benefit with chemotherapeutic agents over pembrolizumab was observed (HR = 0.88, 95% CI 0.45–1.70). Pooled analysis further confirmed that ICIs extended OS solely in EGFR wild‐type patients but not in EGFR mutant patients.

**TABLE 1:** The following key clinical trials report the efficacy of ICIs in EGFR mutant NSCLC patients.

ICI	Clinical trial	Benefit (OS or PFS)	HR (95% CI)	Reference
Atezolizumab	OAK (Phase II; NCT02008227)	No	1.24 (0.71–2.18)	[26]
Nivolumab	CheckMate057 (PhaseII; NCT01673867)	No	1.18 (0.69–2.00)	[27]
Pembrolizumab	KEYNOTE‐010 (PhaseIII; NCT01905657)	No	0.88 (0.45–1.70)	[28]
Pembrolizumab	Phase II; NCT0287994	No	Ceased	[29]
Nivolumab Pembrolizumab Atezolizuma	Pooled analysis (CheckMate057, KEYNOTE010, POPLAR)	No	1.05 (0.70–1.55)	[30]
Nivolumab Pembrolizumab Atezolizumab	Pooled analysis (CheckMate 017, 057, 063, 003)	No	1.11 (0.80–1.53)	[31]

### 3.2 The TME Immunological Scores of EGFR Mutant LUAD Were Lower Than Those of the Wild Type

**FIGURE 1:**
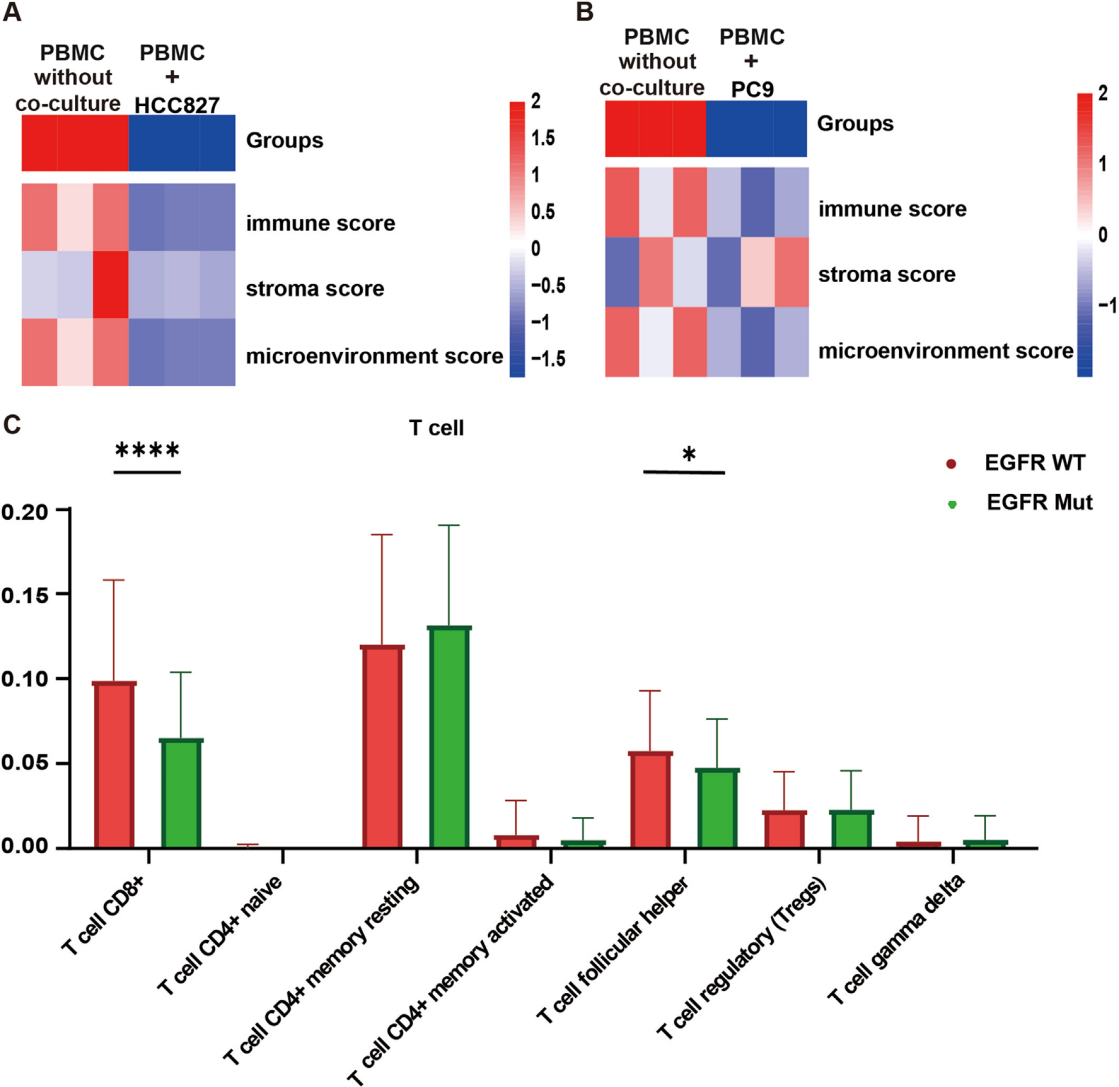
The EGFR mutant cells induced the suppressive immune microenvironment. (A) Upon the co‐cultivation of peripheral blood mononuclear cells with the HCC827 cell line, the transcriptomes were analyzed via high‐throughput sequencing, and the scores of the immune microenvironment were calculated. (B) Upon the co‐cultivation of peripheral blood mononuclear cells with the PC9 cell line, the transcriptomes were analyzed via high‐throughput sequencing, and the scores of the immune microenvironment were calculated. (C) The difference in percentage abundance of tumor‐infiltrating T lymphocytes in EGFR wild‐type and mutant LUAD samples is shown.

TME is highly complex in composition, with low immunogenicity and a non‐inflammatory factor contributing to the limited response to ICIs. To investigate this further, a comparative analysis of immune microenvironment scores was conducted in EGFR wild‐type and mutant LUAD. Transcriptome sequencing of PBMCs co‐cultured with EGFR mutant cells (HCC827 or PC9) was used to calculate immune microenvironment scores compared to the control group (PBMCs without co‐culture). The co‐cultured with EGFR mutant cell groups showed lower immune microenvironment scores than the control group (Figure 1A,B), indicating that EGFR mutant cells can induce an inhibitory immune microenvironment.

TCGA data of EGFR wild‐type and mutant LUAD were analyzed to compare immune cell proportions in the TME. A significant decrease in CD8+ T lymphocytes was found in EGFR mutant LUAD compared to EGFR wild‐type LUAD (*p* < 0.001, Figure 1C). Follicular helper T cells also showed a reduction in EGFR mutant LUAD (*p* < 0.05, Figure 1C).

### 3.3 The Cytotoxicity Scores Were Low in EGFR Mutant LUAD Compared to Wild Type

**FIGURE 2:**
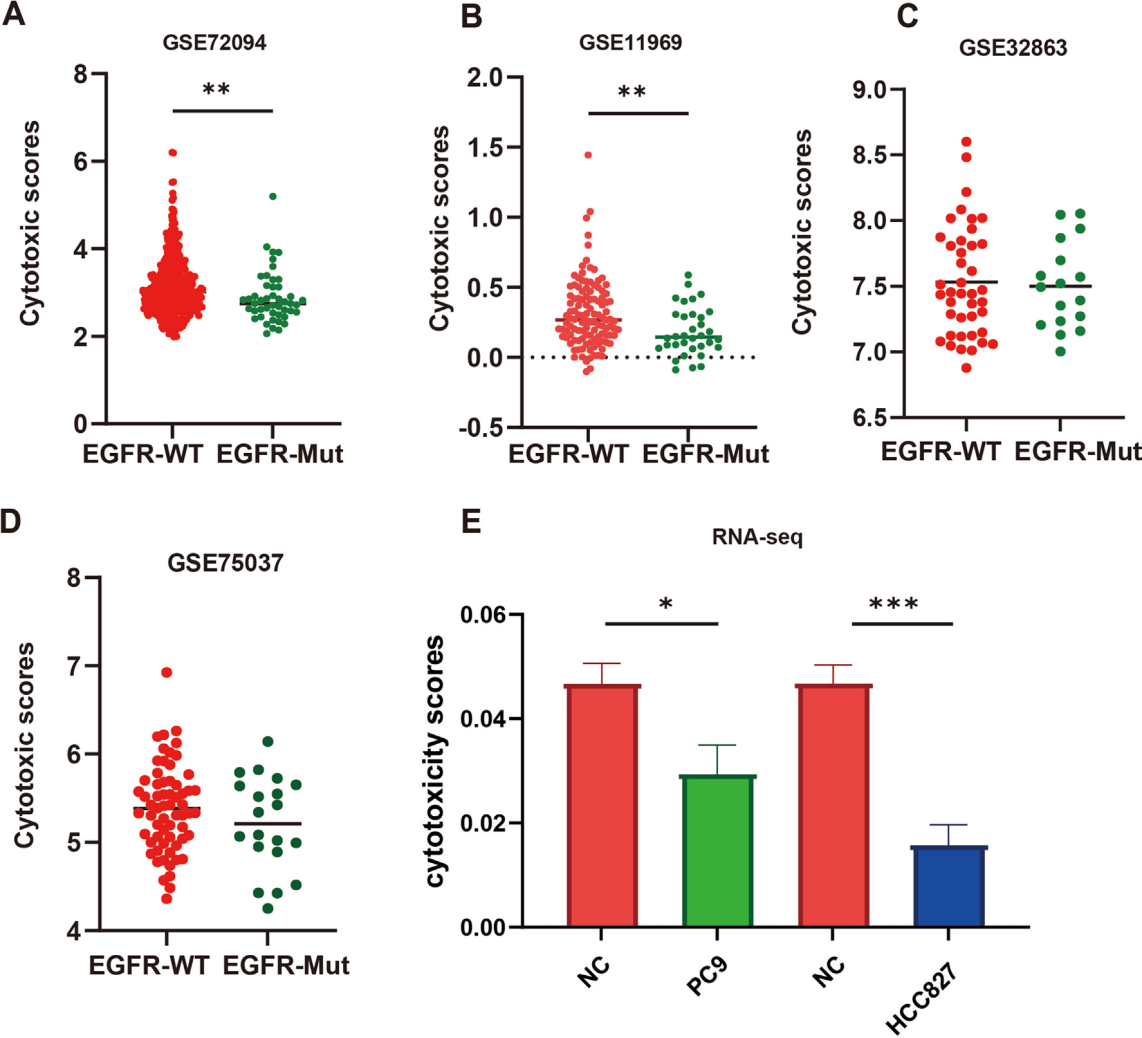
The cytotoxicity scores of immune cells in the tumor microenvironment were low for EGFR mutant LUAD compared to EGFR wild type. (A) GSE72094 showed differences in cytotoxicity scores for EGFR wild‐type and mutant LUAD. (B) GSE11969 EGFR showed difference in cytotoxicity scores between wild‐type and mutant LUAD. (C) Showed difference in EGFR wild‐type and mutant LUAD cytotoxicity scores. (D) GSE75037EGFR showed differences in wild‐type and mutant LUAD cytotoxicity scores. (E) Upon the co‐cultivation of peripheral blood mononuclear cells with the PC9 or HCC827 cell line, the transcriptomes were analyzed via high‐throughput sequencing, and the cytotoxicity scores of the immune microenvironment were calculated. The co‐cultured with EGFR mutant cell line (HCC827 or PC9) group scores were lower than control group.

The study analyzed data from the GEO database to compare cytotoxicity scores between EGFR wild‐type and mutant LUAD. Results of GSE72094 (Figure 2A) and GSE11969 (Figure 2B) showed significantly lower cytotoxicity scores in EGFR mutant LUAD compared to EGFR wild‐type. GSE32863 (Figure 2C) and GSE75037 (Figure 2D) indicated lower cytotoxicity scores in EGFR mutant cases compared to wild‐type, although without statistical significance. Cytotoxicity scores were determined through transcriptome sequencing of PBMC co‐cultured with EGFR mutant cell lines (HCC827 or PC9) versus a control group, revealing lower scores in the co‐cultured group compared to the control group (Figure 2E).

### 3.4 Favorable Outcomes Post‐ICIs Are Linked to Higher Dendritic Cell Infiltration in EGFR Wild‐Type LUAD

**FIGURE 3:**
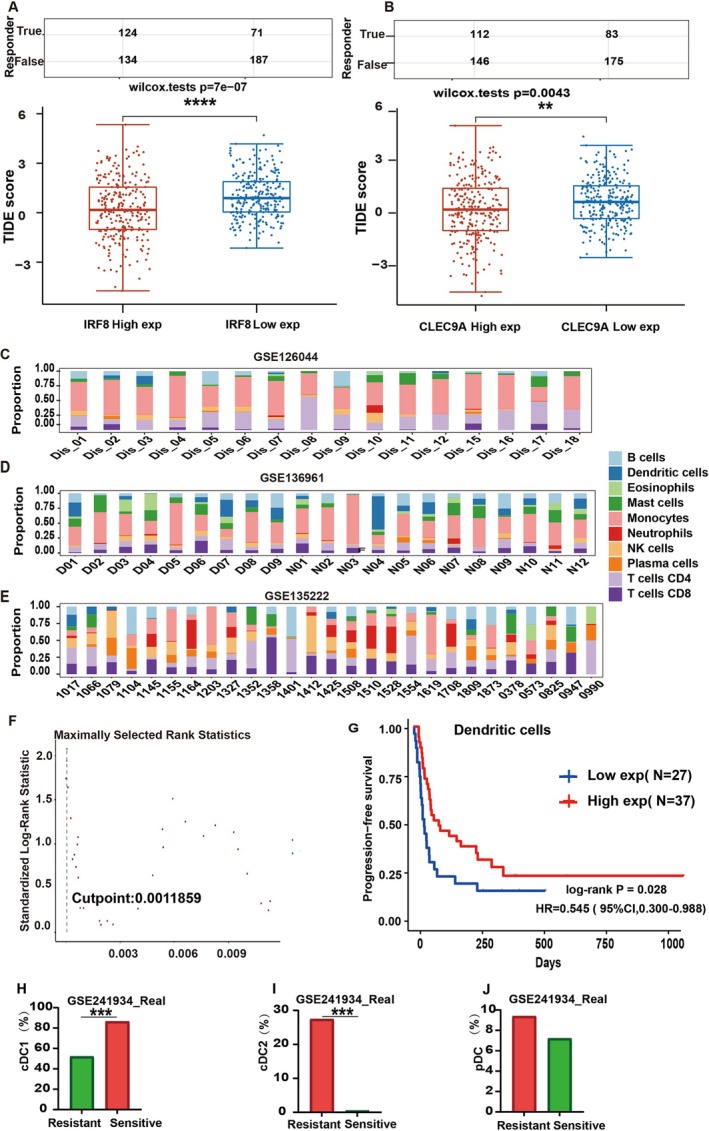
Dendritic cells gene expression signature predicted ICI efficacy. (A) The IRF8 high‐expression group had low TIDE scores, indicating a predicted longer survival period following ICI treatment. (B) The CLEC9A high‐expression group had low TIDE scores, indicating a predicted longer survival period following ICI treatment. (C–E) The stack diagram showed the cell composition of each sample, GSE135222 (*n* = 27), GSE126044 (*n* = 16), and GSE136961 (*n* = 21). Each column in the above figure corresponds to each sample, and a different color indicates the constituent specific gravity of the cells in each sample. The dark blue is the component of DC cells in each sample. (F) The surv_cutpoint value. (G) Kaplan–Meier survival analysis of the different groups of samples from the GEO dataset, patients with high abundance of dendritic cell infiltration had a high therapeutic benefit heel from ICIs. (H) The number of cDC1 in the GSE241934‐Real dataset between the sensitive group and the resistant group following ICIs. (I) The number of cDC2 in the GSE241934‐Real dataset between the sensitive group and the resistant group follwing ICIs. (J) The number of pDC in the GSE241934‐Real dataset between the sensitive group and the resistant group follwing ICIs.

Lower immune scores, reduced CD8+ T lymphocytes, and decreased cytotoxic scores were observed in EGFR mutant LUAD compared to EGFR wild type. DCs are the principal cells involved in the activation of CD8+ T lymphocytes, which are essential for triggering the immune response. The initiation of the immune response is a critical step for ICIs to exert anti‐tumor effects. Therefore, we explored the link between DCs and the anti‐tumor effects of ICIs. We analyzed the relationship between the key molecules expression of DCs and the TIDE score. The TIDE scores were lower in the CLEC9A and IRF8 high‐expression groups compared to the low‐expression groups in LUAD, indicating a predicted longer survival period following ICI treatment (Figure 3A,B).

To investigate the relationship between DCs and ICIs efficacy, we analyzed three datasets (GSE135222, GSE126044, and GSE136961) from the GEO database of patients who underwent immunotherapy. Common gene symbols across datasets were identified, and batch effects were corrected using the removeBatchEffect function. CIBERSORTx was utilized to estimate immune cell composition and quantify the relative levels of different cell types in the mixed cell population (Figure 3C–E). The component ratio of each sample in the datasets and the corresponding clinical tumor progression information were then assessed. The surv_cutpoint function was applied to determine the optimal cutoff point (Figure 3F). Subsequently, 27 cases in the low expression group and 37 cases in the high expression group were identified. Log‐rank analysis revealed a significant difference in survival time between the high‐expression and low‐expression groups of DC markers (*p* = 0.028), suggesting that higher DC gene expression is associated with a more favorable response to ICIs (Figure 3G).

Further analysis in the GSE241934‐Real dataset showed that the number of cDC1 cells was higher in the sensitive group compared to the resistant group and the difference was statistically significant, *p* < 0.001 (Figure 3H), while the number of cDC2 cells was lower in the sensitive group and the difference was statistically significant, *p* < 0.001 (Figure 3I), and the number of pDC cells was also lower in the sensitive group and the difference was not statistically significant (Figure 3J).

### 3.5 The cDC1 Gene Expression Signature Predicted Efficacy of ICIs in LUAD

The GSE241934 dataset showed that the numbers of cDC1, cDC2, and pDC were different in the ICI‐sensitive group and ICI‐resistant group. Therefore, we explored which DC subtype is associated with ICI efficacy.

**FIGURE 4:**
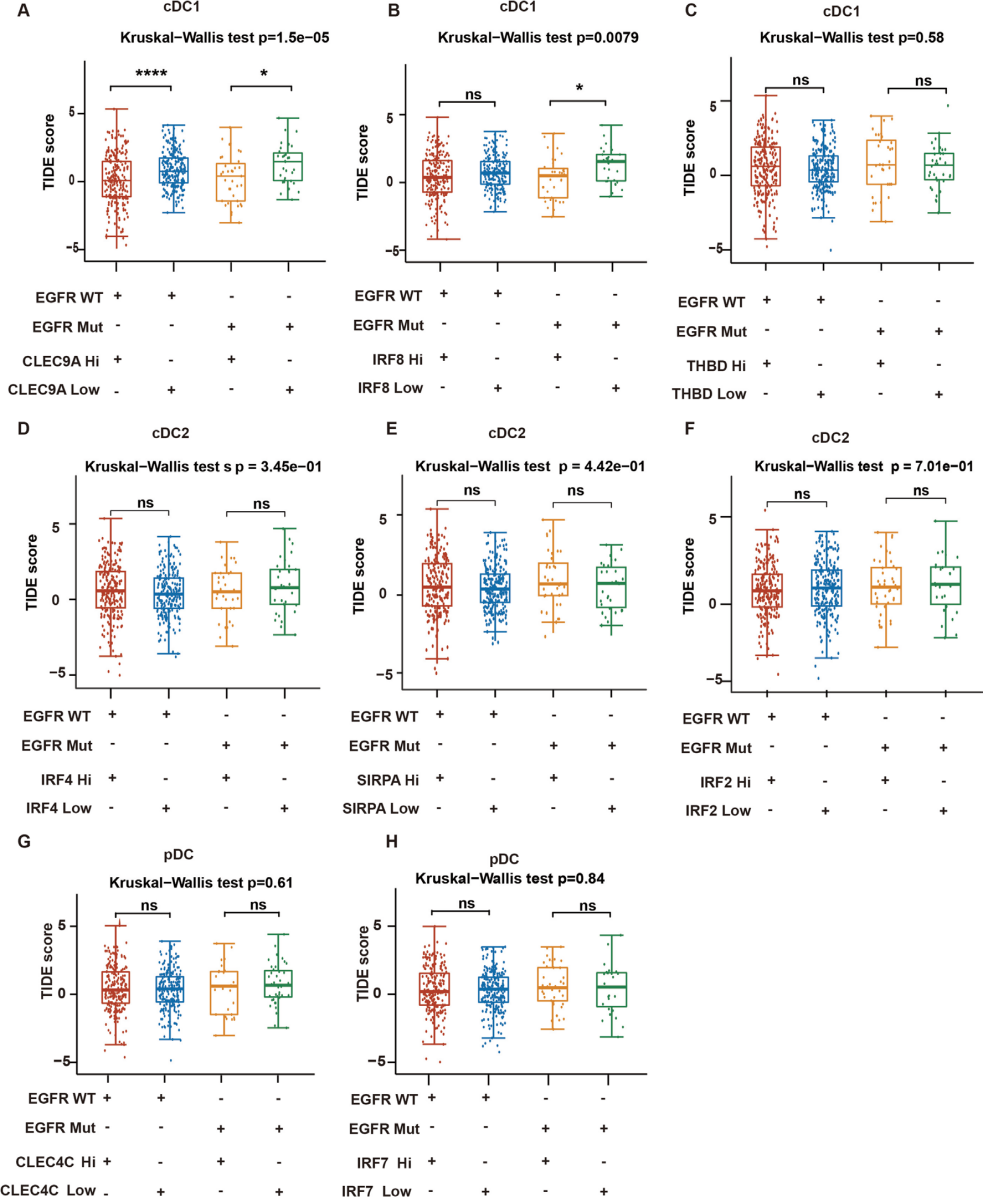
Distribution of TIDE scores in the high‐expression group of LUAD versus low‐expression group of LUAD. First, the cases were divided into EGFR wild type and EGFR mutant groups. Then, we determined a cutoff to classify tumors as the high‐expression group or low‐expression group. We used the average expression of markers across all tumors to determine the expression threshold. (A) TIDE scores in four group divided by the CLEC9A expression and EGFR. (B) TIDE scores of the four groups divided by the IRF8 expression and EGFR. (C) TIDE scores of the four groups divided by the THBD expression and EGFR. (D) TIDE scores of the four groups divided by the IRF4 expression and EGFR. (E) TIDE scores of the four groups divided by the SIRPA expression and EGFR. (F) TIDE scores of the four groups divided by the IRF2 expression and EGFR. (G) TIDE scores of the four groups divided by the CLEC4C expression and EGFR. (H) TIDE scores of the four groups divided by the IRF7 expression and EGFR.

In utilizing the TIDE model for predicting the response to ICIs, a cutoff was established to categorize tumors into high expression and low expression groups. The average expression levels of specific markers (CLEC9A, IRF8, THBD, IRF4, SIRPA, IRF2, CLEC4C, THBD, and IRF7) across all tumors were used to determine the expression threshold. This study investigated the association between the expression of cDC1 molecules (CLEC9A, IRF8, and THBD) and the response to ICIs as indicated by the TIDE score. In EGFR mutant LUAD with high CLEC9A and IRF8 expression, a low TIDE score was associated with a positive ICI outcome (Figure 4A,B). Notably, no statistically significant difference was observed between THBD expression and TIDE score in both EGFR wild‐type and mutant LUAD (Figure 4C). Subsequent analysis examined the impact of ICIs in relation to the expression of cDC2 molecules (IRF4, SIRPA, and IRF2). In both EGFR wild‐type and mutant LUAD, the expression levels of IRF4, SIRPA, and IRF2 were not correlated with the TIDE score (Figure 4D–F). Additionally, the investigation into the effect of ICIs based on the expression of pDC molecules (CLEC4C and IRF7) revealed no significant correlation with the TIDE score in both EGFR wild‐type and mutant LUAD (Figure 4G,H). These findings suggest that cDC1 molecules exhibit a stronger association with the efficacy of immunotherapy in EGFR mutant lung cancer tissues compared to pDC and cDC2.

### 3.6 EGFR Mutant Cells Demonstrated a Strong Ability to Suppress Dendritic Cell Maturation

**FIGURE 5:**
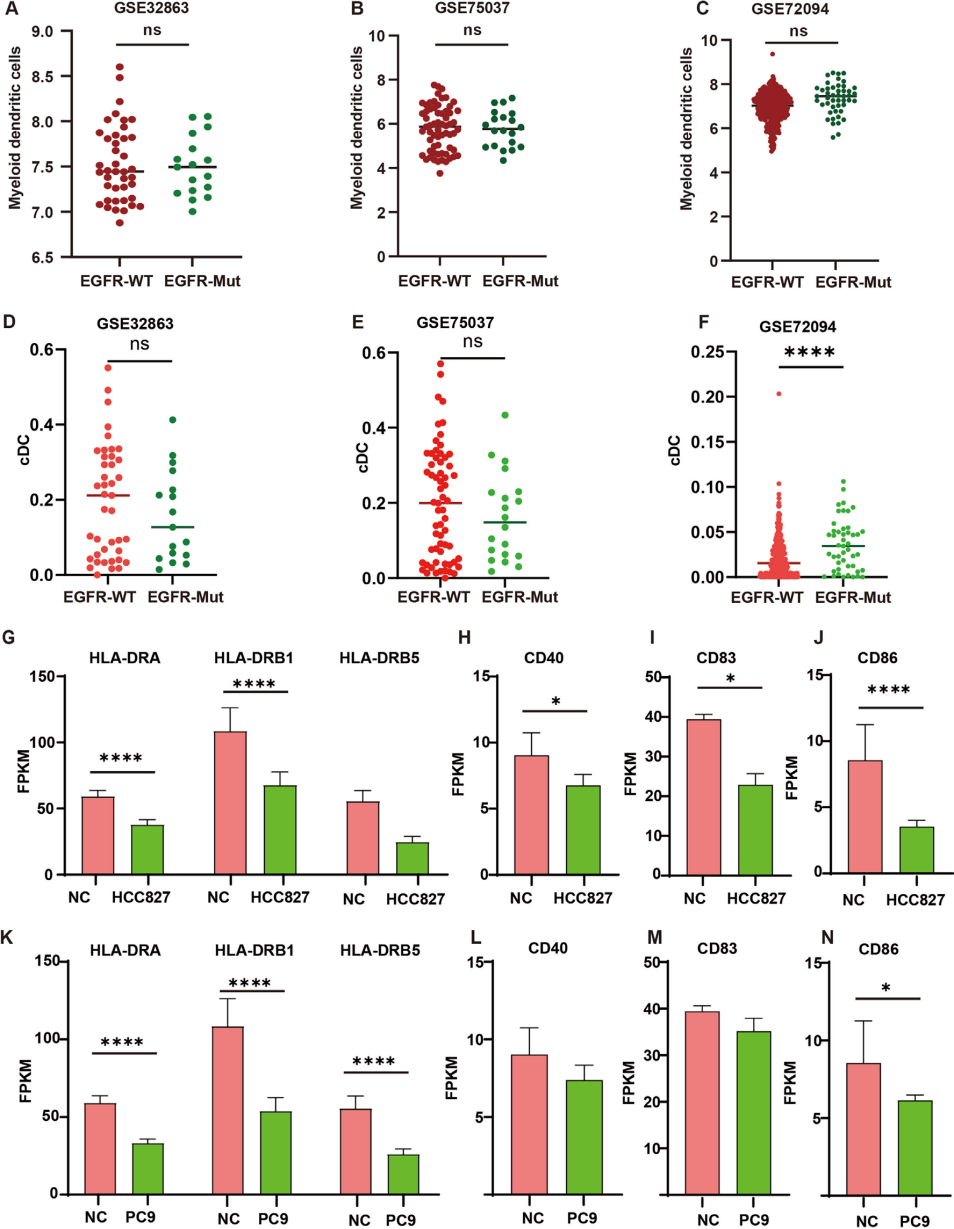
EGFR mutant LUAD cells inhibited DC maturation. (A) Difference in percentage abundance of tumor‐infiltrating DCs in EGFR wild‐type and mutant LUAD was analyzed using GSE32863 data. (B) Difference in percentage abundance of tumor‐infiltrating DCs with EGFR wild‐type and mutant LUAD was analyzed using GSE75037 data. (C) Difference in percentage abundance of tumor‐infiltrating DCs with EGFR wild‐type and mutant LUAD was analyzed using GSE72094 data. (D) Difference in percentage abundance of tumor‐infiltrating cDC in EGFR wild‐type and mutant LUAD was analyzed using GSE32863 data. (E) Difference in percentage abundance of tumor‐infiltrating cDC with EGFR wild‐type and mutant LUAD was analyzed using GSE75037 data. (F) Difference in percentage abundance of tumor‐infiltrating cDC with EGFR wild‐type and mutant LUAD was analyzed using GSE72094 data. (G) HLA‐DR, (H) CD40, (I) CD83, and (J) CD86 mRNA level analysis was performed on cells co‐cultured with HCC827 cells. (K) HLA‐DR, (L) CD40, (M) CD83, and (N) CD86 mRNA level analysis was performed on cells co‐cultured with PC9 cells.

DCs are essential for cytotoxic lymphocyte generation. This study examined DC quantities in EGFR wild‐type and mutant LUAD. Analysis of GSE32863 (Figure 5A), GSE75037 (Figure 5B), and GSE72094 (Figure 5C) datasets indicated no significant decrease in total DCs in EGFR mutant LUAD compared to wild type. Further analysis of cDC numbers in these datasets showed no differences in cDC subpopulations between EGFR mutant and wild‐type LUAD in GSE32863 (Figure 5D), GSE75037 (Figure 5E), and GSE72094 (Figure 5F) datasets.

We investigated the impact of EGFR mutant cells on DC maturation via high‐throughput sequencing. Cells co‐cultured with the HCC827 cells exhibited more pronounced suppression in the expression of HLA‐DR, CD40, CD83, and CD86 than the control group (Figure 5G–J). Furthermore, the results show that cells co‐culturing with the EGFR mutant‐type cell line PC 9 lead to a greater inhibition of HLA‐DR and CD86 expression compared to the control cells (Figure 5K–N).

### 3.7 The Maturation of DCs Was Positively Associated With the Efficacy of Immune Checkpoint Inhibitors

**FIGURE 6:**
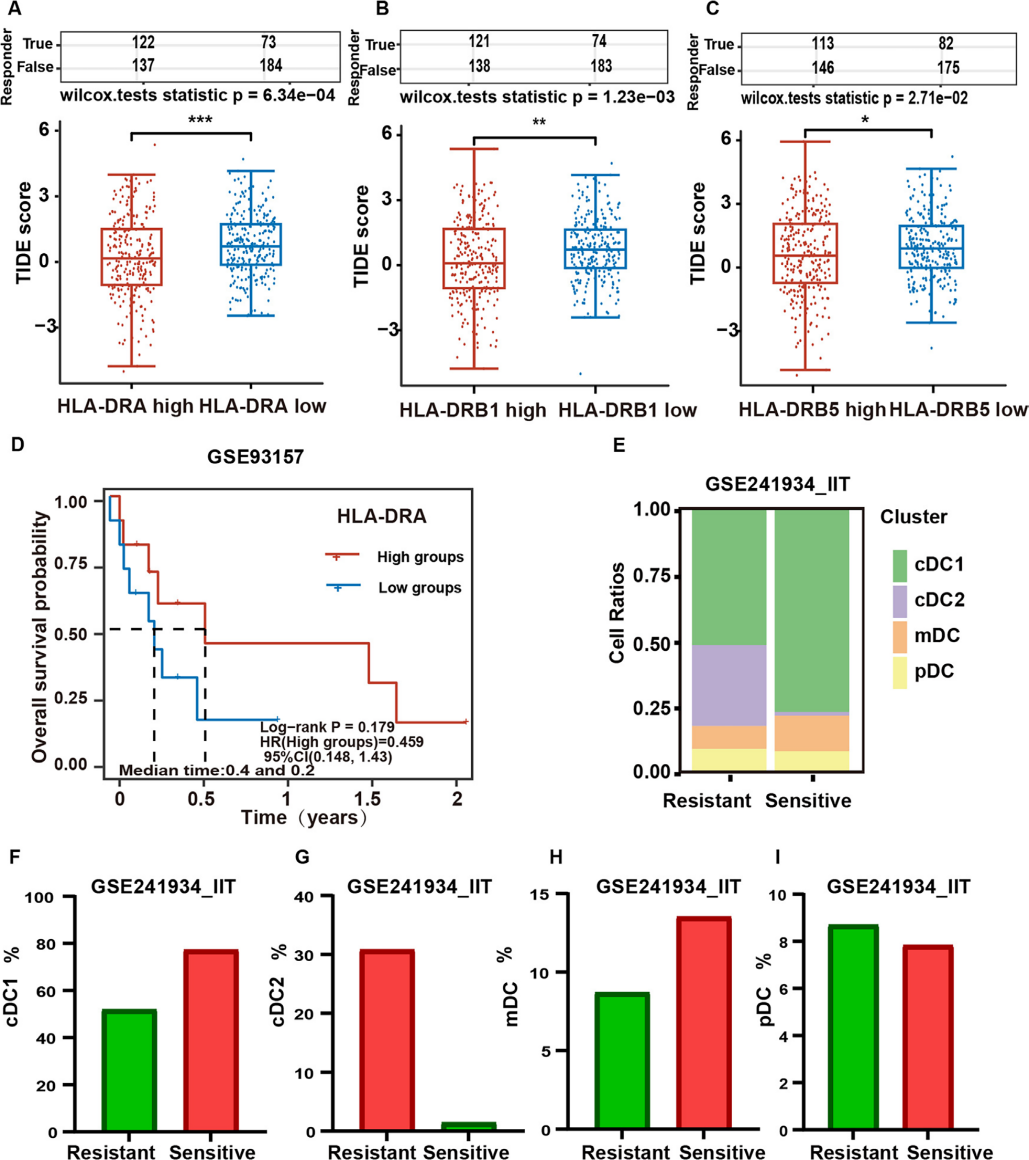
The maturation of DCs has a positive correlation with the efficacy of immune checkpoint inhibitors. (A) TIDE scores of the HLA‐DRA low‐expression group was high. (B) TIDE scores of the HLA‐DRB1 low‐expression group was high. (C) TIDE scores of the HLA‐DRB5 low‐expression group was high. (D) Stratification of cases in GSE93157 according to high and low HLA‐DRA expression levels demonstrated a significantly superior response to ICIs in the high‐expression group as opposed to the low‐expression group. (E) DC subtypes were differ between the ICI‐sensitive and ‐resistant groups in GSE241934‐IIT. (F) The number of cDC1 was high in the ICI‐sensitive group in GSE241934‐IIT. (G) The number of cDC2 was low in the ICI‐sensitive group in GSE241934‐IIT. (H) The number of mature DC was high in the ICI‐sensitive group in GSE241934‐IIT. (I) The number of pDC was low in the ICI‐sensitive group in GSE241934‐IIT.

We found that EGFR mutant cells can inhibit the maturation of DCs and subsequently explored the relationship between DC maturation and ICI efficacy using the TIDE score. The LUAD data were obtained from TCGA and categorized into two groups based on the expression level of HLA‐DR representing maturity: (1) a group with high HLA‐DR expression and (2) a group with low HLA‐DR expression. The group with the lowest HLA‐DRA expression demonstrated the highest TIDE score associated with poor ICI outcomes, as indicated by the Kruskal–Wallis test (Figure 6A). In the low HLA‐DRB1 expression group, a high TIDE score was observed compared to the other groups (Figure 6B), indicating a poor ICI outcome. The low HLA‐DRB5 expression group exhibited a high TIDE score compared to the high HLA‐DRB5 expression groups (Figure 6C), indicating a poor ICI outcome.

The GSE93157 dataset was stratified into high and low HLA‐DRA expression groups. Results revealed that the high HLA‐DRA expression group exhibited a significantly improved response to ICIs compared to the low expression group (Figure 6D), suggesting a crucial role of HLA‐DRA in predicting ICI efficacy.

Subsequent analysis of DC subtypes in the ICI‐sensitive and ‐resistant groups in GSE241934‐IIT showed that mature DCs were more abundant in the ICI‐sensitive group than in the resistant group (Figure 6E,H). Additionally, cDC1 infiltration was significantly higher in the ICI‐sensitive group compared to the resistant group (Figure 6F), while cDC2 and pDC infiltrations were lower in the ICI‐sensitive group than in the resistant group (Figure 6G,I, respectively).

### 3.8 Glycolysis and Oxidative Phosphorylation Exhibited Lower Levels in the ICI‐Sensitive Group of EGFR Mutant LUAD Compared to the Resistant Group

**FIGURE 7:**
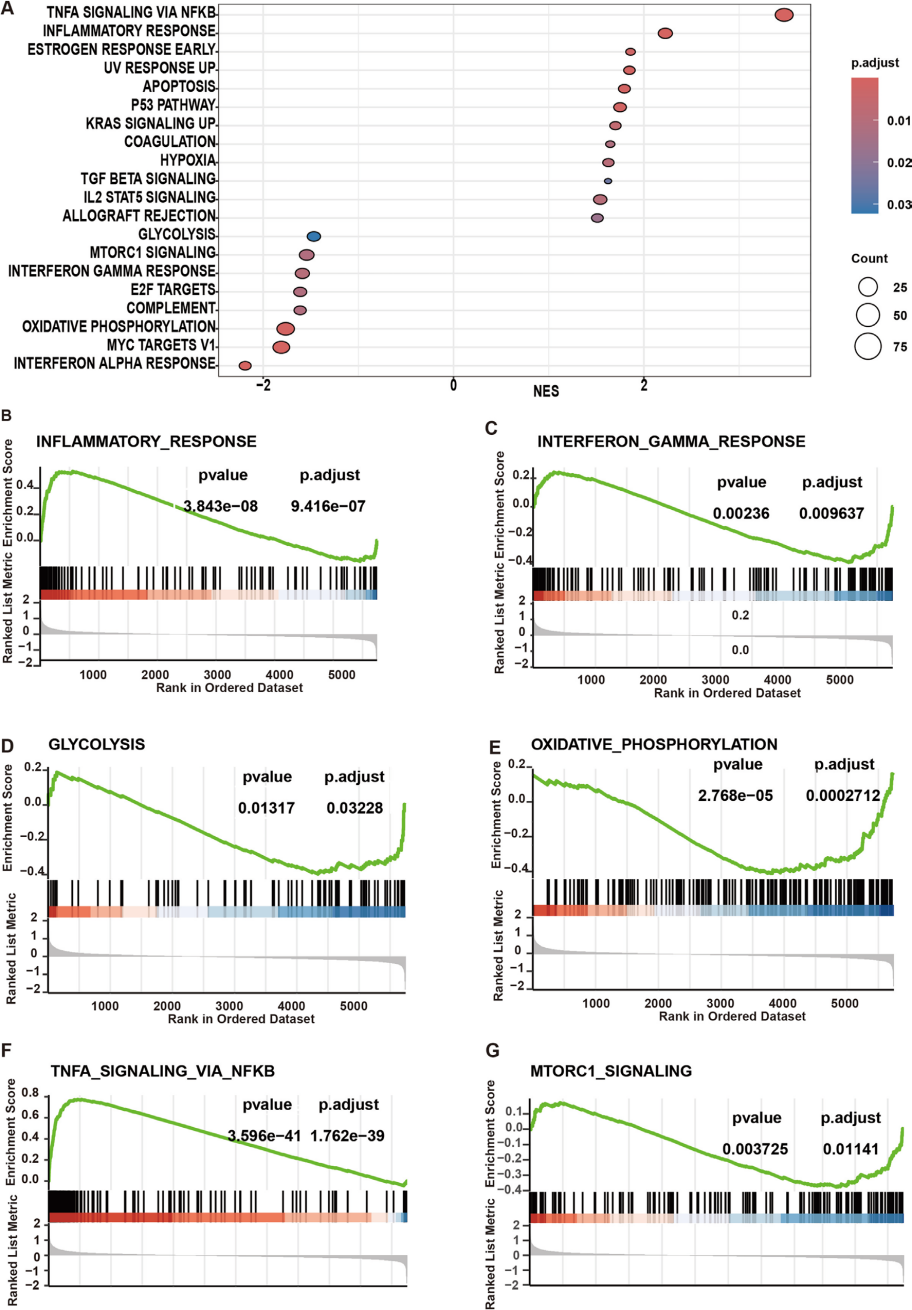
GSEA analysis of cCD1 between ICI‐sensitive and ‐resistant groups in EGFR mutant LUAD. (A) GSEA analysis of all signaling pathways in cCD1 between ICI‐sensitive and ‐resistant groups in EGFR mutant LUAD. (B) GSEA analysis of the INFLAMMATORY_RESPONSE signalling pathway. (C) GSEA analysis of the INTERFERON_GAMMA_RESPONSE signaling pathway. (D) GSEA analysis of the GLYCOLYSIS signaling pathway. (E) GSEA analysis of the OXIDATIVE_PHOSPHORYLATION signaling pathway. (F) GSEA analysis of the TNFA_SIGNALING_VIA_NFKB signaling pathway. (G) GSEA analysis of the MTORC1_SIGNALING signaling pathway.

GSEA analysis of cCD1 was conducted to explore variances in crucial signaling pathways between ICI‐sensitive and resistant groups among patients with EGFR mutations. The results revealed a notable upregulation of the INTERFERON_GAMMA_RESPONSE and INFLAMMATORY_RESPONSE pathways in the ICI‐sensitive cohort, while these pathways were significantly suppressed in the ICI‐resistant group (Figure 7A–C).

In the context of energy metabolism, the ICI‐sensitive group demonstrated significant downregulation in the OXIDATIVE_PHOSPHORYLATION and GLYCOLYSIS signaling pathways, while the resistance group showed marked upregulation (Figure 7D,E). Additionally, the ICI‐sensitive group exhibited elevated TNFA_SIGNALING_VIA_NFKB pathway activity compared to the ICI‐resistant group, which displayed lower activity levels (Figure 7F). Notably, the MTORC1_SIGNALING pathway was notably inhibited in the ICI‐sensitive group, in contrast to its heightened activity in the ICI‐resistant group (Figure 7G).

### 3.9 DCs Genetic Profile Predicted Patient Prognosis

**FIGURE 8:**
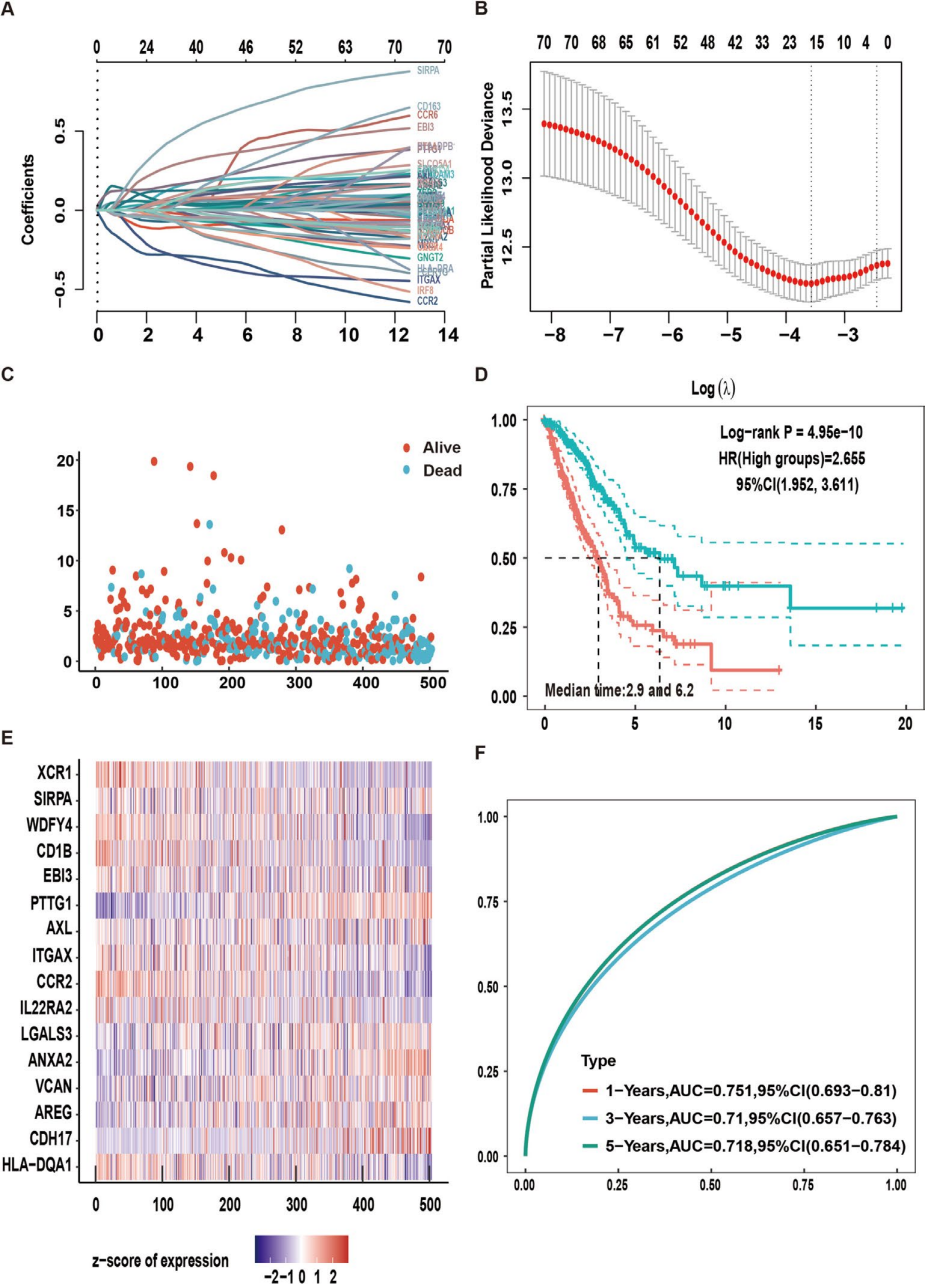
Dendritic cell genetic signature predicted patient prognosis. (A) LASSO coefficient profiles of 89 genes. (B) LASSO regression with 10‐fold cross‐validation obtained 16 prognostic genes using the minimum lambda value. (C) Survival status of the patients. (D) Kaplan–Meier survival analysis of the 16‐gene signature. (E) Heatmap of the expression profiles of the five prognostic genes in low‐ and high‐risk groups. (F) Time‐dependent receiver operating characteristic (ROC) analysis of the five‐gene signature.

Initially, we examine the association between DCs and prognosis. Using LASSO regression with 10‐fold cross‐validation, we identified the optimal lambda value (*λ*_min_ = 0.025) linked to 16 genes that are significantly correlated with OS (Figure 8A,B).

The gene‐based risk score was calculated using Cox coefficients for 16 genes as follows: Riskscore = (−0.0652) * HLA‐DQA1 + (0.0549) * CDH17 + (0.0244) * AREG + (0.057) * VCAN + (0.1279) * ANXA2 + (0.0595) * LGALS3 + (−0.0324) * IL22RA2 + (−0.2136)*CCR2 + (−0.091) * ITGAX + (0.0027)*AXL + (0.1268) * PTTG1 + (0.1107) * EBI3 + (−0.037) * CD1B + (−0.0306) * WDFY4 + (0.1625) * SIRPA + (−0.0346) * XCR1. Subsequently, individual patient risk scores were computed, and the cohort was stratified into high‐risk (*n* = 251) and low‐risk (*n* = 251) groups using the median cutoff point determined with the “survminer” R package. The survival outcomes of all patients in both groups are depicted (Figure 8C), while the prognostic genes are illustrated (Figure 8D). The study utilized the log‐rank test to compare the survival times of patients with high and low expression levels of the DC markers mentioned above. Significant differences in prognosis were observed between the two groups (Figure 8E). Additionally, the 16‐gene prognostic signature demonstrated higher AUC values in a time‐dependent ROC analysis (Figure 8F).

## 4 Discussion

Several clinical studies have shown a poor response of patients with advanced EGFR mutant LUAD to ICIs. The limited predictive accuracy of current biomarkers for ICIs efficacy in these cases remains a significant clinical challenge. Our study found a reduced maturation of tumor‐infiltrating DCs in EGFR mutant LUAD compared to EGFR wild‐type LUAD. In vitro experiments confirmed inhibited DC maturation in EGFR mutant LUAD cells. Analysis using the TIDE score indicated a correlation between DC maturity and ICIs efficacy, suggesting DC maturity as a potential predictive marker and target for regulating ICIs efficacy in EGFR mutant LUAD.

In the clinic, ICIs have made breakthroughs in the clinical treatment of various cancers as the backbone of new oncology therapies. Despite the encouraging outcomes observed with ICIs targeting programmed death receptor 1 (PD‐1) or its ligand (PD‐L1) in clinical trials for various solid tumors, a proportion of patients with high PD‐L1 expression have demonstrated a suboptimal response to such treatments. It is evident that there are inherent limitations in the use of PD‐L1 biomarkers for predicting the efficacy of ICIs [32]. These limitations include variations in PD‐L1 expression cutoff values, the ambiguity of the correlation between PD‐L1 expression and the efficacy of ICIs, and the inconsistency of PD‐L1 expression across different lesions. These uncertainties contribute to the ambiguity of high and low PD‐L1 expression and ICIs efficacy. This suggests that the utilization of tumor cell expression of PD‐L1 as a criterion for determining the appropriateness of ICI therapy may require reconsideration. Another potential biomarker for predicting the efficacy of ICIs is tumor mutational load [33, 34, 35], which, although not suffering from the aforementioned uncertainties, has also been found to be a poor predictor of the efficacy of ICIs in some clinical studies. For example, the KEYNOTE‐158 clinical trial found that TMB was a predictor of PFS and had very little predictive value for OS [36]. These traditional markers have been found to be inadequate in accurately predicting the efficacy of ICIs in the context of advanced LUAD that harbors an EGFR mutation. The aforementioned issues indicate that while immunotherapy shows promise in tumor treatment, its therapeutic impact is limited in individuals with EGFR‐mutated LUAD. The intricate and suppressive TME is a pivotal element in the emergence of drug resistance.

The current study concluded that TME was an important factor affecting the efficacy of ICIs, and the poor efficacy of ICI application in EGFR mutant LUAD was closely related to having a unique immune microenvironment. In this study, using the TCGA database, we explored the difference in the number of CD8+ T lymphocytes in EGFR mutant and wild LUAD and found a significant reduction in the number of CD8+ T lymphocytes in the EGFR mutant LUAD compared with the wild. Concurrently, the capacity of immune cells to eliminate tumor cells within the TME of EGFR mutant LUAD was markedly diminished. Previous studies have similarly identified reduced infiltration of TILs in EGFR mutant tumor tissues [12, 14, 15], which is consistent with the inadequate number of CD8+ T lymphocytes in EGFR mutant LUAD in the present study. Despite the findings of this study and previous studies, which have revealed an insufficient or functionally defective number of CD8+ T lymphocytes in EGFR mutant LUAD, it remains inadequate to fully elucidate the specific mechanism of the poor efficacy of ICIs in EGFR mutant advanced LUAD. Further studies are necessary to elucidate the mechanism. ICIs for EGFR mutant advanced LUAD still require refinement and improvement. Given the therapeutic potential of ICIs that has been demonstrated, but not yet translated into patient benefit, for patients with advanced LUAD harboring EGFR mutation, the urgent need for the exploration of alternative ICI modalities that can benefit a greater number of patients with this condition remains an imperative that researchers must address and continue to pursue.

Tumor cells have been shown to interfere with the process of normal differentiation of DCs, which in turn results in functional defects and reduced numbers of DCs [37, 38]. DCs represent the primary triggering factor for the activation and amplification of CD8+ T lymphocytes. The present study focuses on the differences in the function and number of DCs in EGFR wild‐type and mutant LUAD. We revealed that, while there was no significant difference in the number of DCs in the two types of LUAD, the maturation of DCs was significantly suppressed in the EGFR mutant type. To verify this result, EGFR mutant LUAD cells were co‐cultured with DCs, and the inhibitory effect of EGFR mutant LUAD cells on the maturation of DCs was observed. Furthermore, a comparison of the dataset of neoadjuvant immunotherapy EGFR mutant LUAD revealed a discrepancy in the number of mature DCs between the ICI‐sensitive and ‐resistant groups. Specifically, the ICI‐sensitive group exhibited a higher number of mature DCs, while the ICI‐resistant group demonstrated a lower number of mature DCs. This finding lends further credence to the hypothesis that mature DCs may play a pivotal role in regulating the effectiveness of ICIs. However, to date, no study has directly confirmed the relationship between mature DCs and ICIs, which is a subject that merits further exploration. Despite the evidence from several tumor models, mature DCs are suppressed [16, 17]. However, the findings in studies employing EGFR mutant tumor models remain equivocal. In current clinical practice, the routine performance of quantitative and functional assays of DCs has not been established. Nevertheless, in clinical studies, researchers have begun to employ flow cytometric analyzers to detect the relative proportion of DCs and HLA‐DR expression in the peripheral blood of tumor patients. This approach may emerge as a significant method for predicting the efficacy of ICIs in patients.

A considerable body of research has established that DCs within the TME frequently exhibit signs of dysfunction, encompassing maturation abnormalities and phenotypic shifts. This has been shown to impede their capacity to stimulate CD8+ T lymphocytes, consequently diminishing the efficacy of ICIs therapy. A number of studies have been conducted on the use of DC‐based tumor immunotherapy, both domestically and internationally, with certain achievements being made [18, 19, 20, 21]. These findings provide indirect confirmation of the role of DCs in regulating the efficacy of ICIs. In addition, the present study analyzed the potential predictive value between DC maturation and ICIs using the TIDE score and found that high TIDE scores in EGFR mutant LUAD with low DC maturation suggested poor ICI efficacy. This finding aligns with the observations of the current study, as the ineffective activation of CD8+ T lymphocytes by low DC maturation hinders their expansion and transformation into cytotoxic T lymphocytes, thereby impeding their capacity to exert anti‐tumor effects. This partially elucidates the suboptimal efficacy of ICIs in patients with EGFR mutant LUAD. This partially explains the poor efficacy of ICIs in patients with EGFR mutant LUAD. Consequently, the modulation of DCs is poised to emerge as a pivotal research trajectory for overcoming the resistance of ICIs in patients with EGFR mutant LUAD.

The study is constrained by certain limitations. Initially, the validation of the inhibitory impact of EGFR mutant LUAD on DC maturation relied solely on the co‐culture technique. Furthermore, the specific mechanism driving this effect has not been investigated. Subsequent research endeavors will aim to elucidate this mechanism more comprehensively.

The findings suggest that the EGFR mutant LUAD TME shows impaired DC maturation, reduced CD8+ T cell numbers, and decreased tumor cell elimination ability. Thus, it is proposed that boosting DC maturation could potentially overcome ICI resistance in EGFR mutant LUAD patients.

## Author Contributions

Conceived and designed the experiments, Xiuwen Wang and Yanguo Liu; performed the experiments, Fengqi Xiao; analyzed the data, Fengqi Xiao; contributed reagents/materials/analysis tool, Fengqi Xiao and Yanguo Liu; drafted the manuscript, Fengqi Xiao and Yanguo Liu. All the authors have contributed to the preparation of the manuscript. All the authors have read and approved the final manuscript.

## Conflicts of Interest

The authors declare no conflicts of interest.

## Data Availability

All original data can be obtained by contacting the corresponding author.
